# Supplementary material for: Chromothripsis during telomere crisis is independent of NHEJ, and consistent with a replicative origin
Source: Genome Res. 2019 May;29(5):737–49. doi: 10.1101/gr.240705.118 (PMC6499312; doi:10.1101/gr.240705.118)
Supplement: Supplemental Material [file supp_gr.240705.118_Supplemental_file_1.zip › contigs/annotated_contigs/DB56/contig.2.DB56_length_511_mean_cov_3.81604696673.docx]

**DB56_length_511_mean_cov_3.81604696673**

```
TGCTTCTACAAGAAGCATCTGTGAAGTGTGTACTAAGGCAGGCAGAAAGACCGCTCTTTCTTTTAAGCTCATTTTAAAATCTCAGGTCT
 >chr7:126965819-126966007 - E=2e-101 p=1e-02
CACCACTAATGGCAAGGAAAGGCCAGTTTAAAGCAAGCCAGCTCCTGGCCCCCTACCTAGGTCTTCTCAGGCTGGCACAGAAGTTCCTT

GGCTGGGA|GA|CAAGCAGGTGACAGGCAGGCAGAGAAGGAGAGTGGTCAAGGCCTCTCTGTCATATTTGAATGATGGTATCTCATCTC
         >chr7:121723830-121724155 + E=5e-181
CAATACTGGAAAGAATTTTAGGGTTACCAATGGATTGCTGGCATTGCCCAGGATGGTGGCCCTCATGATTACGAACACTGTGATATCTT

CCATCCTCAAGGAATCCATGGGGATGGCTCCCACTCGCACACCTTGTTGAGACCTTTATTCAATGTTTAAATCTATTAAAATCCTTCAT

TCCTGGATCTATATGTTTGTCAAAAGACTATACTGAAAGCTATAGATCCTAACTGCTGGCCAAAAAAA
```
